# The Role of Tissue and Liquid Biopsy in the Clinical Management of Adult Lung Cancer Patients in King Abdul-Aziz Medical City in Riyadh, Saudi Arabia

**DOI:** 10.7759/cureus.20914

**Published:** 2022-01-03

**Authors:** Lafi Alanazi, Ryan N Alqahtani, Nazish Masud, Meshal M Zuraie, Abdulrahman A Bin Afif, Sulaiman H Alanazi

**Affiliations:** 1 Internal Medicine, College of Medicine, King Saud Bin Abdulaziz University for Health Sciences, Riyadh, SAU; 2 Research, King Abdullah International Medical Research Center, Riyadh, SAU; 3 Research Unit, College of Medicine, King Saud Bin Abdulaziz University for Health Sciences, Riyadh, SAU

**Keywords:** oncology, adenocarcinoma, lung cancer, tissue biopsy, liquid biopsy

## Abstract

Background

Lung cancer is the most fatal malignancy worldwide, characterized by uncontrolled growth in the tissue of the lung(s). The diagnosis of lung cancer depends on the medical history of the patient, along with the physical examination, and various imaging studies. Furthermore, sputum cytology, thoracentesis, or a tissue and liquid biopsy can be examined. The TNM (tumor size, lymph nodes, and metastasis) system is used for staging and grading lung cancer. This study aimed to evaluate the role of tissue vs liquid biopsy in the clinical management of adenocarcinoma, at King Abdulaziz Medical City, Riyadh.

Methods

In this cross-sectional study, all adenocarcinoma patients treated between January 2016 to December 2018 were included using consecutive sampling. The participants were ≥ 18 years old patients with histologically confirmed adenocarcinoma (stage IIIb/IV) regardless of the mutation status. This data was collected through chart review. Data analysis was performed using the IBM Statistical Software for Social Sciences (SPSS) software, version 22 (IBM SPSS Statistics for Windows, Armonk, NY).

Results

A total of 58 participants were included in the analysis. All of them had undergone a tissue biopsy, while only 16 patients underwent liquid biopsy. Out of all patients, 26% of patients had tissue biopsy-related complications (TBRC), with pneumothorax being the most common complication. Single gene testing for epidermal growth factor receptor (EGFR) for patients who underwent tissue biopsy showed a 35% mutation rate. For the anaplastic lymphoma kinase (ALK) gene, 13% were found to be mutated; for the ROS proto-oncogene 1 (ROS1) gene, only 7% were seen to be mutated. For a panel of 12 genes, 25% had the tumor protein 53 (TP53) gene mutation and 39% had the gene Kirsten rat sarcoma viral oncogene homolog (KRAS) mutations. For patients who underwent a liquid biopsy, 20% had the TP53 mutation, 43% had the EGFR mutations on a single gene test and 42% on a panel test, and 10% had the KRAS mutation.

Conclusion

We found that tissue and liquid biopsy showed genetic mutations, particularly with EGFR, TP53, and KRAS genes, among adenocarcinoma patients. Identifying genetic changes in adenocarcinoma patients is essential for charting a targeted therapy. Primary EGFR mutations and rearrangements of ALK or ROS1 are the only gene mutations that can be done with specific tyrosine kinase inhibitors available for clinical practice. Therefore, we recommend further studies to evaluate the role of tissue and liquid biopsy in clinical practice.

## Introduction

Lung cancer has a huge burden of disease and the highest mortality rate among various cancers. In the year 2020, globally, the number of new deaths of lung cancer for both genders was 1,796,144 [1]. Lung cancer or lung carcinoma is malignant and is categorized by uncontrolled growth in the lung tissue(s). Small cell lung cancer (SCLC) and non-small cell lung cancer (NSCLC) are the two main types in which lung cancer is usually grouped. The incidence of NSCLC is higher than SCLC, and both grow differently and, therefore, have different treatments [2].

The tumor, node, and metastasis system (TNM system) is widely used for staging and grading the spread of malignancy. For lung cancer, the first stage is the occult stage where only the sputum shows the cancer cells, while bronchoscopy or imaging tests do not show any tumor in the lung or the size of the tumor is very small. The second stage is stage 0, in which cancer is called carcinoma in situ. The tumor size is very small, and it has not penetrated deeply in tissue or outside of the lungs. The next stage is stage I in which underlying tissues may have cancer, but it has not reached the lymph nodes. Following this, in stage II, nearby lymph nodes also have cancer, along with the chest wall. In stage III, cancer continues to metastasize to the nearby organ(s), such as the esophagus, heart, and trachea. The most advanced stage of lung cancer is stage IV, in which cancer has metastasized to other organs of the body and affects the lung(s) [3].

The types of lung cancer (i.e., SCLC or NSCLC), location, size, and metastasis to other organs determine the method of diagnosis [4]. Physical examination, x-rays, ultrasound, magnetic resonance imaging (MRI), computerized tomography (CT), positron emission tomography (PET), or bone scan can be carried out to investigate for lung cancer. Furthermore, sputum cytology, thoracentesis, or a tissue biopsy can also be conducted [5].

The biopsy of tumor tissue is invasive, and clinical practice often gets affected by its several limitations [6]. It has been demonstrated widely that liquid biopsy can be used as a non-invasive alternative for tissue biopsy by assessing biomarkers specific to tumors. In lung cancer, liquid biopsy has been evolving as a tool for early detection, screening, and monitoring. Revelo et al. conducted a review summarizing all of the biomarkers specific to lung cancer and their essential role in diagnosing lung cancer [7]. When an abnormality is detected by low-dose computed tomography (LDCT), the use of liquid biopsy is carried out as an auxiliary tool to help diagnose lung cancer. Besides, the liquid biopsy also helps detect the reoccurrence of lung cancer by predicting the biomarkers associated with early reoccurrence [8]. Plasma genotyping of solid tumors is also conducted with the help of liquid biopsy [9].

The liquid biopsy is a non-invasive technique that uses circulating biomarkers to provide information regarding the possible presence of cancer. The biomarkers can be protein, deoxyribonucleic acid (DNA), or ribonucleic acid (RNA), and they can be used for diagnosis, detection, and monitoring. Furthermore, even chances of reoccurrences can also be found. It also helps to detect any changes in circulating DNA [10].

It is worth mentioning that tissue biopsy plays a vital role in patient care; however, sometimes it is risky to have an invasive biopsy. It costs more, and it is painful for the patient as well. There are cases in which the tumor is in such a location that it cannot be accessed easily with a needle. In other instances, a patient’s health also does not allow them to undergo such a process. In such circumstances, liquid biopsy is the best alternative. Liquid biopsy detects different proteins, DNA, RNA, exosomes, and sometimes whole cells in various body fluids, including urine, blood, saliva, or cerebrospinal fluid. The accessibility of these body fluids is easy, and a less invasive procedure is required in most cases for the collection of a sample. Another advantage is that it can be repeated easily in comparison to tissue biopsy [11]. 

In this study, we aimed to evaluate the role of tissue and liquid biopsies in the clinical management of adenocarcinoma of the lung (a subtype of NSCLC) and its potential to be a primary diagnostic factor.

## Materials and methods

The cross-sectional study was conducted in the Adult Oncology Department, and the Molecular Oncology Laboratory at King Abdulaziz Medical City, Riyadh from January 2016 till December 2018. We enrolled adult patients with adenocarcinoma of the lung in stages IIIb or IV, regardless of the mutation status, through consecutive sampling techniques.

The departmental and institutional review board (IRB) approval was obtained from King Abdullah International Medical Research Center (KAIMRC) approval #RYD-18-419812-124059. The study maintained the confidentiality and anonymity of the patients and no personal identifiers were included in the analysis. The details of clinical parameters and procedures, like biopsy/treatment, were extracted from the electronic patient files available in the data management system of the hospital. The details related to tissue and liquid biopsy were taken in specific, along with other patient variables (such as age, gender, chronic illnesses, smoking history, immunohistochemistry results, histological subtype, and type of treatment provided).

The main outcome was gene testing details from the tissue and liquid biopsies. The tissue biopsy was performed for all 58 patients, while the liquid biopsy was ordered for only 16 patients. All specimens were sent to the oncology laboratory for next-generation sequencing (NGS) and other molecular assays either as a panel or a single gene testing. For tissue biopsy, the gene sequencing was performed as a panel for all the patients, whereas for liquid biopsy, only specific gene markers were identified and performed based on the recommendations of the treating doctor. The results were reported back to the treating physicians for consideration in the management protocol. Data from molecular studies included information on the presence of wild and mutated genes, as well as treatment plans.

The data was initially extracted and entered in Microsoft Excel sheets, version 2016 (Microsoft® Corp., Redmond, WA) and subsequently transferred to the IBM Statistical Software for Social Sciences (SPSS) software, version 22 (IBM SPSS Statistics for Windows, Armonk, NY) for analysis. The descriptive statistics for all of the variables were reported and summarized as tables and figures. Categorical variables were reported as frequency and percentages out of the total, while the continuous variables were reported as means with standard deviations (SD). The grouping variable was tissue vs liquid biopsy. The results for the presence of wild and mutated genes were reported as frequency and percentages for both types of biopsies. 

## Results

**Figure 1:**
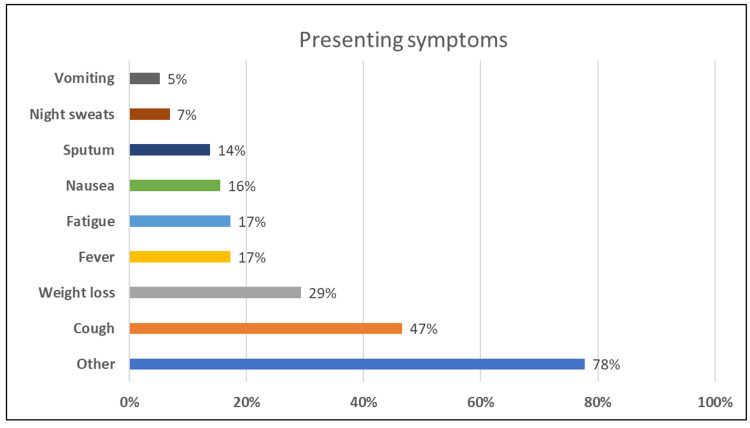
Presenting symptoms of lung cancer patients (N = 58)

This study enrolled 58 patients with adenocarcinoma of the lung from January 2016 to December 2018, of whom about two-thirds were male and one-third were female with a mean age of 68 ± 11.4 years. Most of the patients were classified as stage IV, and more than half of the patients have died. Among them, 60% of patients were currently smoking and 22% had a history of smoking. The most-reported comorbidities were diabetes and hypertension, and nearly half of our patients were affected. One-third of patients (n = 17) also suffered from shortness of breath (Table 1). Among the presenting symptoms, the cough was reported by 47%, followed by weight loss at 29%, and fatigue and fever each reported at 17% by our patients (Figure 1). 

**Table 1:** Profile of Lung Cancer Patients (n = 58) SD: standard deviation

Variables	Categories	Frequency	Percentage
Age (years)		Mean ± SD = 68 ± 11.4	
Gender	Male	38	66%
	Female	20	34%
Marital Status	Single	1	2%
	Married	44	76%
	Widow	11	19%
	Unknown	2	3%
Occupational Status	Unemployed	8	14%
	Employed	5	8%
	Retired	23	40%
	Homemaker	17	29%
	Unknown	5	9%
Patient Status	Alive	28	48%
	Dead	30	52%
Stage of Cancer	Stage 3b	3	5%
	Stage 4	55	95%
Diabetes	No	30	52%
	Yes	28	48%
Hypertension	No	27	47%
	Yes	31	53%
Ischemic heart disease	No	55	95%
	Yes	3	5%
Shortness of breath	No	41	71%
	Yes	17	29%
Allergy	No	29	50%
	Yes	5	9%
	Unknown	24	41%
Past surgical history	No	35	60%
	Yes	12	21%
	Unknown	11	19%
Family history of cancer	No	35	60%
	Yes	3	5%
	Unknown	20	35%
Current smoking status	No	22	38%
	Yes	35	60%
	Unknown	1	2%
History of smoking	No	44	76%
	Yes	13	22%
	Unknown	1	2%

The gene testing of tissues and liquid biopsies was performed either as a single gene test or as a panel as per the doctor’s order. A tissue biopsy was ordered and performed for all the participants (n = 58). Conversely, the liquid biopsy was ordered and performed for only 16 of the 58 participants (n = 16). For a liquid biopsy, single-gene or panel testing was not ordered by default for all patients who underwent tissue biopsy. For the EFGR gene, single-gene test results showed about 35% of patients had positive mutations in tissue biopsy and 43% in liquid biopsy; in panel testing, liquid biopsy showed a higher positivity than tissue biopsy with 42% vs 21% reported mutations, respectively. Also, regarding the gene Kirsten rat sarcoma viral oncogene homolog (KRAS) in panel testing, 39% of patients had positive mutations in tissue biopsy and 10% in liquid biopsy. In the liquid biopsy, 20% of patients who had undergone panel testing had positive mutations in the TP53 gene and 25% in tissue biopsy. Anaplastic lymphoma kinase (ALK), and ROS proto-oncogene 1 (ROS1), gene testing did not show positive mutation for any patient who had undergone liquid biopsy. RET proto-oncogene gene testing was not ordered for any of the sixteen patients who underwent liquid biopsy (Tables 2-3). 

**Table 2:** Comparison of Gene Testing Results of Tissue Versus Liquid Biopsy *Wild is considered as a normal gene **The single or the NGS panel is not ordered as default for all the genes the ordering doctor selects the gene(s) for testing because of cost involved. Each gene was analyzed as a separate yes/no variable; the total might not add up to n = 16 for the individual frequencies. ALK: anaplastic lymphoma kinase; BRAF: proto-oncogene B-Raf; EGFR: epidermal growth factor receptor; ERBB2: erythroblastic oncogene B 2; KRAS: the gene Kirsten rat sarcoma viral oncogene homolog; MAP2K1: mitogen-activated protein kinase kinase 1; MET: mesenchymal-epithelial transition; NGS: next-generation sequencing; NRAS:  neuroblastoma ras viral oncogene homolog; PIK3CA: phosphatidylinositol-4,5-bisphosphate 3-kinase catalytic subunit alpha; RET: RET proto-oncogene; ROS1: ROS proto-oncogene 1, receptor tyrosine kinase; TP53: tumor protein 53

		Tissue biopsy (N = 58)				Liquid biopsy** (n = 16)			
Panel Type	Gene types	Wild*		Mutated		Wild*		Mutated	
		N	%	N	%	N	%	N	%
Single gene	EGFR	32	65%	17	35%	4	57%	3	43%
	ALK	28	88%	4	13%	…	…	…	….
	ROS1	26	93%	2	7%	…	…	…	….
NGS panel	EGFR	19	79%	5	21%	7	58%	5	42%
	ALK	22	96%	1	4%	10	100%	0	0%
	ROS1	22	96%	1	4%	10	100%	0	0%
	KRAS	14	61%	9	39%	9	90%	1	10%
	NRAS	16	100%	0	0%	10	100%	0	0%
	BRAF	22	96%	1	4%	10	100%	0	0%
	ERBB2	22	96%	1	4%	10	100%	0	0%
	MAP2K1	17	100%	0	0%	10	100%	0	0%
	MET	20	91%	2	9%	10	100%	0	0%
	PIK3CA	15	94%	1	6%	10	100%	0	0%
	TP53	15	75%	5	25%	8	80%	2	20%
	RET	20	95%	1	5%	…	…	…	….

**Table 3:** Profile of Molecular Testing CD2: cluster of differentiation 2; CD5: cluster of differentiation 5; CK7: cytokeratin 7; TTF1: thyroid transcription factor-1

Variables	Categories	Frequency	Percentage
Tissue biopsy (n = 58)	Not done	0	0%
	Performed	58	100%
Liquid biopsy (n = 58)	Not done	42	72%
	Performed	16	28%
Next-generation sequencing (n = 57)	Not done	35	61%
	Done	22	39%
Immunohistochemistry (IHC)			
CK7 (n = 32)	Negative	1	3%
	Positive	31	97%
CD5 (n = 7)	Negative	5	71%
	Positive	2	29%
CD2 (n = 5)	Negative	5	100%
	Positive	0	0%
Napsin (n = 37)	Negative	7	19%
	Positive	29	81%
TTF1 (n = 53)	Negative	10	19%
	Positive	43	81%

All adenocarcinoma patients required a tissue biopsy, of which 26% of patients had tissue biopsy-related complications presenting as a pneumothorax. Chemotherapy was given to 97% of adenocarcinoma patients who were supposed to start it as treatment, as well as 91% who received radiotherapy and 94% who were on target treatments (Table 4).

**Table 4:** Complications and Treatment Summary (n = 58)

Variables	Categories	Frequency	Percentage
TBRC (tissue biopsy-related complications) (n = 58)	No	43	74%
	Yes	15	26%
Pneumothorax (n = 58)	No	43	74%
	Yes	15	26%
Hemorrhage (n = 58)	No	58	100%
	Yes	0	0%
Chemotherapy (n = 31)	Not done	1	3%
	Done	30	97%
Radiotherapy (n = 23)	Not done	2	9%
	Done	21	91%
Chemoradiotherapy (n = 2)	Not done	1	50%
	Done	1	50%
Target treatment (n = 16)	Not done	1	6%
	Done	15	94%

## Discussion

Lung cancer is the most common type of fatal malignancy globally, and its diagnosis is based on various tools, including patient history, medical examination, imaging, biomarkers, and various types of biopsy. In this study, we focused on the role of tissue and liquid biopsy in the clinical evaluation of adenocarcinoma of the lung, particularly the non-small cell category of lung cancer. Liquid biopsy is based on analyzing tumor material that is released into the circulation. Liquid biopsy can be complementary to a tissue biopsy, for both primary diagnosis and in-progress evaluation, especially in the detection of somatic genetic changes. Identifying molecular aberrations in key elements of the signal transduction pathways involved in tumor growth and survival, called gene-dependent tumors, has dramatically changed the approach to treating lung cancer.

Mutations in the epidermal growth factor receptor (EGFR) and TP53 genes are the most common and clinically essential targets. In patients with alterations in the aforementioned genes, tyrosine kinase inhibitors (TKIs) have shown significant results and have become the standard for treatment. For the treatment of advanced NSCLC, EGFR TKIs (gefitinib, erlotinib, afatinib, osimertinib) and ALK inhibitors (crizotinib, ceritinib, and alectinib) have been approved in Europe and the United States [12]. The majority of adenocarcinomas have at least one driving mutation that could be the target of treatment, and this has led to the initiation of many clinical trials for new targeted therapies [13]. These targeted therapies have shown substantial clinical benefits, with high response rates and a better quality of life for patients. Different TKIs have reported objective response rates (ORRs) of 60% - 70% and disease control rate (DCR) of up to 80% - 90% [14]. Other examples of carcinogenic drivers for adenocarcinoma include modifications in protein kinase B (AKT), BRAF, human epidermal growth factor receptor-2 (HER2), phosphatidylinositol-4,5-bisphosphate 3-kinase catalytic subunit alpha (PIK3CA), MET, ROS1, RET, and KRAS [15].

Our study had predominantly male participants, even though the incidence of lung cancer has relatively increased internationally among females [16-17]. As our study focused specifically on stages IIIb and IV adenocarcinoma of the lung, this could be the reason for differences in the overall higher percentage of males in our population. The majority of our participants were current smokers and 22% had a history of smoking in past as well. This finding is in line with a previous study that reported a strong association between cigarette smoking and adenocarcinoma [18]. Apart from other symptoms, the cough was seen in (47%), followed by weight loss (29%) of our patients. Another study conducted at Jedahh reported a slightly higher percentage of cough among (76%) of their patient sample. As the Jeddah study was a prospective study and patients were followed this could be one of the reasons for the difference in the overall percentage of the symptoms in comparison to our study [19].

Regarding tissue biopsy results, in our study, 32 patients underwent a single gene test for EGFR, and it was found that 35% had mutated genes and 65% had normal genes. In comparison, liquid biopsy showed a higher positivity for mutations in a single EGFR gene test and panel testing with 43% and 42%, respectively. The study conducted in China with 747 patients showed a slightly higher mutation with an overall EGFR mutation rate of 50% vs active EGFR mutation rate of 48% among their stage IIIb/IV lung adenocarcinoma patients [20].

Also, regarding the KRAS gene in panel testing in our study, 39% of patients had positive mutations in tissue biopsy and 10% in liquid biopsy. Twenty percent of patients with liquid biopsy and 25% with tissue biopsy underwent panel testing and had positive mutations in the TP53 gene. It was seen that the majority of these alterations were not specific to lung cancer. This limitation makes tissue biopsy necessary, at least in the initial diagnosis. Regardless, liquid biopsy can still give vital assistance, especially when used in combination with tissue sampling, to the treating physician and the pathologist to better characterize individual tumors, even in the setting of limited tissue. Regarding tissue biopsy, we reported that pneumothorax is a frequent complication related to tissue biopsy. Moreover, sometimes, due to poor physical condition or inaccessible location, tissue biopsy is not safely obtainable [11]. 

Liquid biopsy, which aims to look for neoplastic gene changes in blood samples, has currently been applied to EGFR-mutated NSCLC and is under investigation for NSCLC positive for ALK and ROS1. Multiple methods are available for target detection of individual modifications or broad-spectrum analyses of ctDNA. Meanwhile, other circulating materials (circulating tumor cells (CTCs), extracellular vesicles (EVs), or cell-free microRNAs (cfmiRNAs) from cancer cells are being investigated. However, the current data and technology make us assume that the replacement of tissue biopsy with liquid biopsy is unlikely to be seen in a few years. To date, liquid biopsy has a complementary role to tissue biopsy. Despite many advantages, the liquid biopsy may not be able to fully replace its tissue counterpart in detecting clinically relevant mutations in patients with advanced adenocarcinoma. However, it may be a useful tool when making treatment decisions.

There were some limitations to our study. First, a liquid biopsy was not requested for all patients included in the study. The high cost of the liquid biopsy was the main reason why the treating doctors had little or no choice when ordering a liquid biopsy; thus, this limitation was beyond the control of the research team. The comparison between the detection of mutated gene detection of tissue vs liquid could not be performed due to the small sample in the liquid biopsy group. Therefore, advanced statistical analysis was not reported in the current study, which was one of the major limitations to study findings. Additionally, the insufficient and missing data in the electronic medical record system was a major challenge for the data collection team, so all the variables of interest could not be reported as planned. Furthermore, the mortality analysis of the patients was not performed as it was beyond the scope of the current study, and we only focused on the tissue and liquid biopsy reporting for the gene sequencing. 

Future studies are required with a larger sample size to look into the diagnostic efficacy of tissue vs liquid biopsy gene testing. Although liquid biopsy has the potential to be the preferred tool in the future, tissue biopsy is the current gold standard and cannot be overlooked. Therefore, future research might help to find a clear conclusion on the said debate between tissue vs liquid biopsy testing.

## Conclusions

In conclusion, this study manifested a higher prevalence of adenocarcinoma of the lung(s) in men. The most common symptoms reported in this study were sputum production and cough. Pneumothorax was detected as a common complication associated with tissue biopsy. Identifying genetic changes in adenocarcinoma patients is essential for charting a targeted therapy. The results of tissue testing and liquid biopsies showed multiple genetic mutations, especially in the EGFR, KRAS, and TP53 genes. Primary EGFR mutations and rearrangements of ALK or ROS1 are the only genes that can be tested with specific tyrosine kinase inhibitors available for clinical practice. Therefore, we recommend that further studies be done to evaluate the role of tissue and liquid biopsy in clinical practice.
